# Duration of Breastfeeding and Risk Reduction of Breast Cancer among Mothers Who Have Ever Breastfed: A Case-Control Study Conducted in Bahir Dar, Ethiopia

**DOI:** 10.1155/2024/1987378

**Published:** 2024-09-21

**Authors:** Fentanesh Nibret Tiruneh, Peter Austin Morton Ntenda

**Affiliations:** ^1^ Department of Applied Human Nutrition Faculty of Chemical and Food Engineering Bahir Dar Institute of Technology Bahir Dar University, Bahir Dar, P.O. Box 79, Ethiopia; ^2^ Bahir Dar Food and Nutrition Research Center Bahir Dar Institute of Technology Bahir Dar University, Bahir Dar, P.O. Box 79, Ethiopia; ^3^ Malaria Alert Centre Kamuzu University of Health Sciences, Private Bag 360, Chichiri, Blantyre 3, Malawi

## Abstract

**Introduction:**

Breast cancer is currently the most frequently detected cancer in women and the primary cause of cancer-related deaths globally. The incidence of breast cancer has significantly increased in countries across sub-Saharan Africa, counting Ethiopia. There are multiple determinants of breast cancer, a few of these can be changeable whereas others are not. Evidence suggests that breastfeeding, which is a changeable determinant, reduces breast cancer risk. However, there is a lack of evidence specifically linking the duration of breastfeeding to breast cancer risk. To date, no study has been conducted on the association between the duration of breastfeeding and the likelihood of breast cancer among Ethiopian women.

**Objective:**

The aim of this study was to evaluate the relationship between breastfeeding duration and breast cancer risk in Ethiopian mothers who had breastfed, taking into account other significant determinants.

**Methods:**

A hospital-based case-control study was carried out in Bahir Dar, Ethiopia, involving 203 women (70 cases and 133 controls). Face-to-face interviews were performed using a standardized, validated questionnaire that assessed various sociodemographic, reproductive, lifestyle, and dietary characteristics. Differences between cases and controls were evaluated using the chi-square test. The associations among factors were examined through bivariate and multivariable binary logistic regression, with results presented as odds ratios and 95% confidence intervals.

**Results:**

The multivariable investigation revealed that an inverse relationship between breastfeeding duration and breast cancer risk. Mothers who breastfed for a longer period had a 93% lower risk of breast cancer (AOR = 0.07; 95% CI: 0.021–0.21) compared to those who breastfed for a shorter duration. Younger mothers had a 95% lower likelihood of developing breast cancer (AOR = 0.05; 95% CI: 0.003–0.91) than older mothers. Additionally, mothers with sedentary behaviour were 10.53 times more likely to develop breast cancer (AOR = 10.53; 95% CI: 5.21–21.36) than those who were moderately or highly active. Mothers who experienced chest therapy were 6.43 times more likely to develop breast cancer (AOR = 6.43; 95% CI: 3.20–13.90) compared to those who had not.

**Conclusions:**

Interventions such as breastfeeding counselling and promoting the recommended duration of breastfeeding are crucial in minimizing the risk of breast cancer. Enhancing physical activity should also be viewed as a vital approach for lowering breast cancer risk. Additionally, healthcare professionals need to limit exposure to chest radiation therapy to reduce the likelihood of breast cancer.

## 1. Introduction

Breast cancer is the most frequently detected cancer among females globally and the primary reason of cancer-related mortalities [1]. Alarmingly, one in eight women worldwide will be diagnosed with breast cancer at some point in their lives [2]. Sub-Saharan African countries have experienced a significant rise in breast cancer cases, marked by high death-to-incidence ratios resulting from late-stage diagnoses and inadequate access to actual treatments [3]. Breast cancer in Ethiopia exhibits a concerning trend with a continuous and rapid increase in incidence rates over the years. It is the most common cancer, making up 33% of cancers in females and 23% of all cancers [4]. The reasons behind this rising incidence are not fully understood, although reproductive and lifestyle factors are likely contributors [5]. Notably, breast cancer in Ethiopia often occurs at a younger age compared to Western countries and frequently presents at a more progressive stage. Although there are several recognized determinants for breast cancer, including family history, early onset of menstruation, late menopause, adult height, and reproductive history, these factors are difficult to change [6].

Breastfeeding holds significant relevance in the prevention of breast cancer as it represents a modifiable risk factor. While not yet fully comprehended, several molecular explanations have been hypothesized to elucidate the preventive effect of breastfeeding on breast cancer risk. Breastfeeding has been associated with hormonal changes and alterations in molecular histology within the breast, which may help lower an individual's likelihood of developing breast cancer [7, 8]. The protective effects of breastfeeding have been observed across various countries and ethnic groups, indicating that the safeguarding mechanism is attributed to biological changes in the breast rather than environmental or socioeconomic factors [9, 10]. In addition to reducing the breast cancer risk, breastfeeding provides numerous other health benefits for mothers. It has been linked to a reduced likelihood of developing endometrial and ovarian cancers, as well as a lower chance of chronic conditions that are risk factors for cancer, including hypertension, type 2 diabetes, and obesity [11–13]. Moreover, breastfeeding provides numerous benefits for infants and young children, helping to decrease the risk of diarrhea, ear infections, and lower respiratory tract infections. Furthermore, breastfeeding is linked to a reduced risk of sudden infant death syndrome, diabetes, asthma, and childhood obesity [14]. Breastfeeding offers a protective effect against breast cancer regardless of the number of births, with a notably stronger impact observed in women who breastfeed for 12 months or more. Studies indicate that worldwide breastfeeding rates already prevent more than 20,000 breast cancer deaths annually, and this number could be further reduced by an additional 20,000 if breastfeeding duration increased to 12 months per child in developed countries [15, 16].

Despite the significant benefits of breastfeeding in reducing the breast cancer risk and promoting overall health for both mothers and children, breastfeeding duration in urban areas of Ethiopia has been experiencing a notable decline, falling below the standards set by the World Health Organization (WHO). The WHO advices exclusive breastfeeding for the first six months, and encourages continuing breastfeeding along with introducing suitable complementary foods for up to two years or beyond [17]. Although almost all Ethiopian mothers (97%) initiate breastfeeding at some point [18], the duration of breastfeeding decreases significantly, with rates dropping from 74% to 64% for infants aged 0-1 month and from 74% to 36% for infants aged 4-5 months [19]. Several factors contribute to the decline in breastfeeding duration. Perceived insufficient milk supply, the burden of work, lack of support, returning to work, unsupportive working environments, and the prospect of getting pregnant again are among the reasons why mothers choose not to breastfeed or discontinue breastfeeding earlier than recommended [20, 21].

As a result of limited treatment options and unfavorable prognoses, a majority of females in Ethiopia are detected with advanced-stage of breast cancer [3]. Consequently, primary prevention measures, such as encouraging longer breastfeeding practices, are essential and cost-effective strategies for lowering cancer incidence and mortality rates in developing countries like Ethiopia, where breast cancer incidence is increasing and mortality rates remain high. Notably, there is a lack of prior studies exploring the link between breastfeeding duration and breast cancer risk among Ethiopian women. Therefore, this study aims to investigate the relationship between breastfeeding duration and breast cancer risk among women in Ethiopia. We hypothesize that a longer duration of breastfeeding may be linked to a lower risk of breast cancer.

## 2. Methods

### 2.1. Study Design and Participants

A hospital-based case-control study was carried out at Felege Hiwot Comprehensive Specialized Hospital (FHCSH) in Bahir Dar, Ethiopia, from May 16 to July 16, 2021. In the first phase, 70 cases were identified, consisting of women with pathologically confirmed breast cancer referred to the chemotherapy clinics at FHCSH. These women served in place of the study cases. In the second phase, 133 control participants were selected from women admitted to other departments of the same hospital, who were diagnosed with non-neoplastic illness not associated with long-term lifestyle modifications. These control participants were included to compare the factors between with cases. Selecting both groups from the same hospital ensures similar healthcare-seeking behaviors and access to medical services, controlling for environmental and healthcare-related variables. By choosing women with non-neoplastic diseases not influenced by long-term lifestyle modifications, minimize confounding factors.

### 2.2. Data Collection

Demographic and socioeconomic information was collected through self-reporting, including factors such as education level, place of residence, and monthly income. Detailed data on health history, family history of breast cancer, reproductive risk factors (age at menarche, menopause status, age at first pregnancy, breastfeeding history, and duration breastfeeding for each live birth, use of modern contraceptives), and family history of other cancers were also obtained. Anthropometric measurements, such as weight and height, were collected, and body mass index (BMI) was computed from these measures (kg/m^2^). Participants' physical activity levels were assessed and categorized as sedentary, light activity, moderate activity, and very/extremely active. Dietary patterns were evaluated, distinguishing between healthy and unhealthy patterns. A healthy dietary pattern involved the intake of fruits, vegetables, and whole grains, while limiting processed foods, red meat, and sweetened drinks. Conversely, an unhealthy dietary pattern included the ingesting of fatty and greasy foods, sugary foods, strongly salted foods, and excessive alcohol intake.

### 2.3. Statistical Analysis

Data were analyzed using SPSS software, version 20. To assess differences between cases and controls, the chi-square test was employed. The results of these tests were presented as frequencies and percentages, providing a clear depiction of the distribution of sociodemographic, reproductive health-related, behavioral, and disease-related characteristics among the study participants.

To examine the associations between predictor variables and the outcome variable, bivariate binary logistic regression was conducted. This analysis identified variables that were significantly related with the outcome by calculating odds ratios (OR) along with their 95% confidence intervals (CI). Predictors with a *P* value below 0.25 in the bivariate analysis were considered for inclusion in the subsequent multivariable analysis. The threshold of *P* < 0.25 was chosen to ensure that potentially important variables were not excluded prematurely.

A multivariable binary logistic regression model was subsequently used to evaluate the combined impact of multiple predictor variables on the outcome variable. This method controls for confounding factors and provides adjusted odds ratios (AOR) with 95% confidence intervals, offering a more precise estimate of the relationship between predictor and outcome variables.

The goodness-of-fit for the logistic regression model was assessed using the Hosmer–Lemeshow test and the Akaike Information Criterion (AIC). A higher *P* value from the Hosmer–Lemeshow test indicates a better fit of the model. All the models had *P* values greater than 0.05, suggesting they were a good fit. The Akaike Information Criterion (AIC) is another metric used to assess the model fit, where a lower AIC value indicates that the model is a better fit.

(1) $$\begin{array}{c} \text{AIC}=2k-2\;\ln\left(L\right), \end{array}$$

where *L* represents the maximized likelihood of the model, which measures how well the model fits the data. The term *k* represents the number of parameters in the model, including the intercept and any additional predictors.

### 2.4. Ethical Issues

Ethical approval for this study was approved by the Ethics Committee of the Bahir Dar Institute of Technology (BiT). Informed consent was obtained from all participants before their involvement, following a thorough explanation of the study's objectives. Participants were assured of the confidentiality of their identities and the information they provided. They were also given the opportunity to ask questions about the study and were informed of their right to withdraw or discontinue participation at any time.

## 3. Results

### 3.1. Baseline Characteristics

Table 1 presents the distribution of various characteristics among individuals with breast cancer cases and those in the control group. The majority (94.9%) of controls were aged 18–29 years. For the age groups 30–49 years and above 50 years, cases were more prevalent 40.9% and 42.9% respectively (*P* ≤ 0.001). Married women were more common among controls (71.6%) than cases 28.4% (*P*=0.078). Educational status revealed significant differences (*P*=0.004), with illiterate women more prevalent among cases (45.7%) than controls (54.3%), and those with college education or above being significantly more among controls (83.3%) compared to cases (16.7%). Occupational status also showed significant differences (*P*=0.008), with self-employed women and those in government workers being more frequent among controls (72.7% and 74.5%) compared to cases (227.3% and 25.5%) respectively. Higher income brackets had more controls compared to cases (*P*=0.01).

Modern contraceptive use was significantly higher among controls (69.4%) compared to cases (30.6%) (*P*=0.033). History of childbirth was more common among controls (65.5%) compared to cases (34.5%) (*P*=0.087). Among those who had given birth, age at first childbirth showed significant differences (14–18 years had more controls (54%) compared to cases (46%) (*P*=0.041). Breastfeeding duration showed a highly significant difference, with less than or equal to a year being more common among cases (73.1%) compared to controls (29.9%), and >1 year being significantly higher among controls (78.8%) compared to cases (21.2%) (*P* ≤ 0.001). Premenopausal women were more common among controls (68.5%) compared to cases (31.5%), (*P*=0.249).

Table 1 also shows that 8.1% of breast cancer patients were at Stage I, 31.4% were at Stage II, 27.9% are at Stage III, and 32.6% are at Stage IV.

Chest radiation therapy was significantly higher among cases (70.0%) compared to controls (30.0%), (*P* ≤ 0.001). Physical activity levels showed significant differences, with sedentary behavior being more common among cases (80.0%) compared to controls (20.0%), and higher levels of physical activity more common among controls (light activity (71.2%) compared to cases (28.8%) and moderate and above activity (73.7%) compared to cases (26.3%), (*P* ≤ 0.001)). Dietary patterns did not show significant differences, with healthy dietary patterns being slightly more common among controls (62.3%) compared to cases (37.7%), (*P*=0.274). Nutritional status (BMI) showed highly significant differences, with being underweight was more common among cases (90.5%) compared to controls (9.5%), (*P* ≤ 0.001). Normal was more common among controls (69.1%) compared to cases (30.9%), and overweight was only observed in controls (100%).

### 3.2. Bivariate Analyses

Table 2 outlines the determinants linked to breast cancer risk. The bivariate analysis, demonstrated that multiple variables were significantly associated with breast cancer risk. These variables included duration of breastfeeding, age, residence, education level, occupation, monthly income, age at first birth, chest radiation therapy, and physical exercise status.

Notably, mothers who breastfed for greater than 1 year had a 90% lower odds of breast cancer than those who breastfed for 1 year or less (COR = 0.10, 95% CI: 0.05–0.21). Younger mothers aged 18–29 years had an 85% lower odds of breast cancer than those older than 50 years (COR = 0.15, 95% CI: 0.06–0.35). Educational status also played a crucial role, as mothers who were illiterate had 6.87 times greater odds of breast cancer than those with college and above education (COR = 6.87, 95% CI: 3.10–15.27). Similarly, mothers with primary and secondary education had 3.83 and 4.10 times greater odds of breast cancer, than college and above educational level, respectively. Occupation was another contributing factor, with housewives having 3.73 times greater odds of breast cancer than government employees (COR = 3.73, 95% CI: 1.79–7.40). Regarding monthly income, mothers with a monthly income of ≤1000 ETB had 5.54 times greater odds of breast cancer than those with >6000 ETB (COR = 5.54, 95% CI: 2.02-15.17).

Furthermore, the bivariate analysis revealed that mothers with a sedentary lifestyle had 17.65 times greater odds of breast cancer than to those who were moderately or highly active (COR = 17.65, 95% CI: 5.87–23.06). Mothers who received chest radiation therapy had 6.07 times higher odds of breast cancer than to those who did not (COR = 6.07, 95% CI: 2.81–13.08).

In the bivariate analysis, the interaction term for sedentary and lightly active individuals with breastfeeding duration showed a statistically significant (COR = 0.79; 95% CI: 0.37−0.17 and COR = 0.05; 95% CI: 0.20−0.15)), indicating a protective effect in relation to breast cancer. The interaction term for those who had chest radiation therapy and breastfeeding duration also showed statistically significant (COR = 0.09; 95% CI: 0.05–0.19), indicating a very strong protective effect.

### 3.3. The Multivariable Analyses

The multivariable analysis results showed that some factors were statistically linked to breast cancer risk. These factors include the duration of breastfeeding, age, physical activity level, and exposure to chest radiation therapy.

Mothers who breastfed their child for over a year experienced a 93% lower risk of developing breast cancer than those who breastfed for one year or less (AOR = 0.07; 95% CI: 0.021–0.21). Additionally, participants in the younger age group had significantly reduced odds of developing breast cancer than those in the older age group (AOR = 0.05; 95% CI: 0.003–0.91).

Mothers with sedentary behavior and light physical exercise had significantly higher chance of developing breast cancer than their counterparts who were moderately or highly active. Specifically, mothers with sedentary behavior had 53% higher odds (AOR = 10.53; 95% CI: 5.21–21.36), while those with light physical exercise had 6.13 times higher odds (AOR = 6.13; 95% CI: 1.042–16.00).

Furthermore, mothers who had previously experienced chest radiation therapy were 43% more likely to develop breast cancer compared to those who had not undergone such therapy (AOR = 6.43; 95% CI: 3.20–13.90).

While the bivariate analyses showed significant protective effects of breastfeeding duration in relation to physical activity and chest radiation therapy, these effects were not statistically significant when adjusted for other variables in the multivariable analyses. Nonetheless, the direction of the association still suggests a protective effect.

## 4. Discussions

This study examined the relationship between breastfeeding duration and the breast cancer risk among mothers who had breastfed their child/children. To our knowledge, this is the first study in Ethiopia to investigate the relationship between breastfeeding duration and breast cancer risk, considering other significant factors.

In line with previous studies conducted in other countries and consistent with our hypothesis [15, 22–26], this study showed an inverse relationship between the duration of breastfeeding and breast cancer risk. Mothers who breastfed their child/children for over a year experienced a 93% reduction in the chance of developing breast cancer than those who breastfed for less than or equal to a year.

Various mechanisms have been suggested to account for these observed relationships. Firstly, breastfeeding has been found to lower levels of hormones like estrogen and progesterone, which are associated with the development of breast cancer. Secondly, the lactating mother's shorter menstrual cycle, resulting from various hormonal changes during breastfeeding, limits exposure to these hormones, thus breastfeeding helps prevent the development of cancer cells. Thirdly, it encourages the shedding of breast tissue, which assists in removing cells that may have DNA damage. Fourthly, longer breastfeeding duration is linked to lower concentrations of harmful organochlorines in breast tissue. Finally, during lactation, a hormonally regulated negative growth factor is expressed in human breast cells, which further contributes to the protective effect against breast cancer [27–30].

The lower breast cancer risk linked with breastfeeding duration remains consistent across high-income and low-income countries, regardless of factors such as age, menopausal status, ethnic group, or age at first birth. The evidence suggests that breastfeeding consistently lowers breast cancer risk [31], indicating that the protective effect is likely due to biological changes in the breast rather than environmental or socioeconomic factors [10]. Breastfeeding has proven to be the most effective method for protecting mothers against breast cancer. Consequently, interventions like breastfeeding counselling and improved programs are essential for lowering breast cancer risk. These initiatives offer important support and education to encourage breastfeeding and its benefits, ultimately enhancing the well-being of both mothers and their infants.

Among the covariates examined, age, physical activity status, and chest radiation therapy were found to have a statistically significant association with breast cancer. The study found that younger mothers had a reduced likelihood of developing breast cancer than older mothers. It is well established that increasing age is the strongest risk factor for invasive breast cancer, and evidence consistently shows that breast cancer risk rises with age [32]. It is reported that as women age, their hormone levels change specifically, estrogen levels tend to increase during perimenopause and decrease after menopause. Thus, higher levels of estrogen over a longer period of time can contribute to rise breast cancer risk [32]. Therefore, the findings of this study align with existing knowledge regarding age as a significant factor in breast cancer risk.

This study found a significant link between physical activity status and breast cancer likelihood. Women who engaged in sedentary behavior were found to have a higher chance of rising breast cancer development than those who were moderately or vigorously active. These findings are consistent with previous studies that have demonstrated a lower breast cancer risk among individuals who engage in regular exercise [33–35].

Therefore, enhancing physical activity should be acknowledged as a crucial strategy for lowering breast cancer risk. Exercise may contribute to this reduction in several ways. Firstly, regular physical activity helps reduce body fat, which can subsequently lower levels of hormones like estrogen that may promote breast cancer cell growth [36]. Additionally, exercise is known to boost the immune system and decrease inflammation, both of which could influence the development and progression of cancer [37, 38]. These mechanisms highlight the importance of incorporating physical activity into one's lifestyle as a means of reducing breast cancer risk.

In line with earlier studies [39, 40], this study also revealed that individuals who had experienced chest radiation therapy were at a higher risk of developing breast cancer compared to those who had not experienced such treatment. Furthermore, a history of chest X-ray radiation exposure was positively correlated with breast cancer risk. Radiation can directly damage the DNA double helix, triggering damage response mechanisms that lead to cell death, necrosis, and disruptions in normal cell division. These disruptions can subsequently alter the biological traits of cancer cells [40]. The findings highlight the importance of considering the potential risks associated with chest radiation therapy and its impact on breast cancer development. Efforts should be made to minimize unnecessary radiation exposure and carefully evaluate the risks versus benefits of using radiation in medical treatments.

The higher percentages of breast cancer cases in Stages II, III, and IV indicate that many breast cancer patients present with more advanced cancer, which may reflect either a later diagnosis or a tendency for the disease to progress more rapidly in this cohort. The distribution highlights the need for early detection and intervention strategies to manage and potentially reduce the number of patients diagnosed at more advanced stages.

In interpreting the results of this study, it is important to acknowledge potential limitations. One limitation is the relatively small sample size, which may affect the generalizability of the findings. Since the study is an unmatched case-control study, the control group, consisting of women with non-neoplastic diseases, might differ from the case group of women with breast cancer in demographic factors such as age, socioeconomic status, or health behavior. These differences could affect the study's outcomes, leading to inaccurate comparisons. While efforts were made to include all eligible cases admitted to the study hospital, there is a possibility that some breast cancer patients who were undiagnosed may have been excluded. This could potentially impact the representativeness of the study population and limit the generalizability of the results. To mitigate selection bias, controls were selected from women admitted to the same hospital, but for noncancerous conditions. However, it is important to recognize that the hospital-based study design may introduce inherent biases and may not fully capture the broader population.

## 5. Conclusion

Based on the findings of this study, which demonstrated an inverse association between breastfeeding duration and breast cancer risk, it becomes evident that breastfeeding is a valuable modifiable risk factor for breast cancer. Promoting breastfeeding through proper education and support not only enhances infant health but also has the potential to reduce the burden of breast cancer. Our study underscores the importance of primary prevention efforts, with a focus on educating women about the significance of breastfeeding for extended periods in line with the recommendations of the WHO to decrease the risk of breast cancer.

Besides emphasizing the benefits of breastfeeding, the current study also underscores the significance of moderate to vigorous physical activity in lowering breast cancer risk. Promoting regular exercise among women can positively influence breast cancer prevention. Additionally, healthcare providers should be cautious with chest radiation therapy, as the study findings suggest that such exposure is linked to an increased risk of breast cancer. It is crucial to minimize unnecessary radiation exposure to reduce breast cancer risk.

Overall, while this study emphasizes the importance of primary prevention strategies, including promoting breastfeeding, encouraging physical activity, and exercising caution with chest radiation therapy, it is crucial to consider the potential limitations. These include the small sample size and potential selection bias, which may affect the findings' validity. Despite these limitations, our study provides valuable insights that can inform public health strategies to effectively reduce breast cancer risk.

## Figures and Tables

**Table 1:** Characteristic of ever breastfed mothers among cases and controls in in Felege Hiwot Comprehensive Hospital, Bahir Dar, Ethiopia (*n* = 203).

	Category	Control (%)	Case (%)	*X* ^2^ (*P* value)
*Sociodemographic factors*				
Residence	Urban	77 (70.6)	32 (29.4)	0.066
	Rural	56 (59.6)	38 (40.4)	
Age	18–29 years	37 (94.9)	2 (5.1)	≤0.001
	30–49 years	68 (59.1)	47 (40.9)	
	>50 years	28 (57.1)	21 (42.9)	
Marital status	Married	111 (71.6)	44 (28.4)	0.078
	Unmarried	22 (45.8)	26 (54.2)	
Educational status	Illiterate	50 (54.3)	42 (45.7)	0.004
	Primary	29 (67.4)	14 (32.6)	
	Secondary	9 (63.3)	5 (35.7)	
	College and above	45 (83.3)	9 (16.7)	
Occupation	House wife	39 (52.0)	36 (48.0)	0.008
	Self-employed	56 (72.7)	21 (27.3)	
	Government	38 (74.5)	13 (25.5)	
Religion	Orthodox	124 (63.9)	70 (36.1)	0.055
	Others	9 (100)	0 (0)	
Monthly income	≤1000	8 (38.1)	13 (61.9)	0.010
	1001–3000	33 (61.1)	21 (38.9)	
	3001–6000	49 (67.1)	24 (32.9)	
	>6000	43 (78.2)	12 (21.8)	

*Reproductive health related factors*				
Age of menarche	≤15	83 (66.9)	41 (33.1)	0.595
	>15	50 (63.3)	29 (36.7)	
Modern contraceptive	Yes	109 (69.4)	48 (30.6)	0.033
	No	24 (52.2)	22 (47.8)	
Childbirth history	Yes	133 (65.5)	70 (34.5)	0.087
	No	40 (76.9)	12 (23.1)	
Age at the time of first childbirth (*n*n = =208)	14–18	34 (54.0)	29 (46.0)	0.041
	19–24	77 (68.8)	35 (31.2)	
	≥25	22 (78.6)	6 (21.4)	
How many children do you have? (*n*n = =208)	1–3	75 (52.2)	40 (34.8)	0.918
	≥4	58 (65.9)	30 (34.1)	
How long breastfeed your children	≤1 year	14 (29.9)	38 (73.1)	≤0.001
	>1 year	119 (78.8)	32 (21.2)	
Menopause status	Premenopause	87 (68.5)	40 (31.5)	0.249
	Postmenopause	46 (60.5)	30 (39.5)	

*Behavioral and disease related characteristics*				
Chest radiation therapy	Yes	9 (30.0)	21 (70.0)	≤0.001
	No	124 (71.7)	49 (28.3)	
Stages of cancer	Stage I		4 (5.7)	
	Stage II		22 (31.4)	
	Stage III		20 (28.5)	
	Stage IV		24 (32.3)	
Physical activity	Sedentary	5 (20.0)	20 (80.0)	≤0.001
	Light activity	86 (71.2)	35 (28.8)	
	Moderate and above activity	42 (73.7)	15 (26.3)	
Dietary pattern	Healthy	76 (62.3)	46 (37.7)	0.274
	Unhealthy	57 (70.4)	24 (29.6)	
Nutritional status (BMI)	>18.5 kg/m^2^	2 (9.5)	19 (90.5)	≤0.001
	18.5–24.9 kg/m^2^	114 (69.1)	51 (30.9)	
	≥>=25.0 kg/m^2^	17 (100)	0 (0%)	

**Table 2:** Bivariate and multivariable analyses to examine the duration of breastfeeding and its relation with the breast cancer risk among mothers who had breastfed in Bahir Dar, Ethiopia, 2021 (*n* = 203).

Factors	Category	COR with 95% CI	AOR with 95% CI
Duration of breastfeeding	>1 year	0.10 (0.05–0.21)^∗∗^	**0.07 (0.021–0.21)** ^∗^
	≤1 year	1	1

Age	18–29 years	0.15 (0.6−0.35)^∗∗^	**0.05 (0.003–0.91)** ^∗^
	30–49 years	0.89 (0.47–1.70)	2.09 (0.36–9.30)
	>50 years	1	1

Residence	Urban	0.72 (0.43–1.21)^∗^	1.33 (0.32–5.45)
	Rural	1	1

Marital status	Married	0.65 (0.40–1.18)	
	Unmarried	1	

Educational status	Illiterate	6.87 (3.10–15.27)^∗∗^	1.10 (0.78–5.69)
	Primary	3.83 (1.56–9.40)^∗∗^	8.58 (0.63–11.71)
	Secondary	4.10 (1.49–11.27)^∗∗^	5.50 (0.39–7.78)
	College and above	1	1

Occupation	Housewife	3.73 (1.79–7.40)^∗∗^	0.55 (0.06–5.27)
	Self-employed	1.56 (0.78–3.14)^∗=∗^	0.65 (0.09–4.97)
	Government emp.	1	1

Monthly income	≤1000	5.54 (2.02–151.7)^∗∗^	2.10 (0.24–8.35)
	1001–3000	1.98 (0.92–4.24)^∗^	0.74 (0.12–4.70)
	3001–6000	1.73 (0.81–3.67)^∗^	0.99 (0.22–4.49)
	>6000	1	1

Dietary patterns	Healthy	0.74 (0.43–1.27)	
	Unhealthy	1	

Total number of children born	0	4.6 (0.27–8.28)	
	1–3	1.15 (0.64–1.2.05)	
	4 and above	1	

Age at first childbirth (*n* = 208)	14–18	2.60 (0.97–6.95)^∗^	3.86 (0.33–4.48)
	19–24	1.50 (0.58–3.80)	5.70 (0.64–5.69)
	25 and above	1	1

Physical activity level	Sedentary	17.65 (5.87–23.06)^∗∗^	**10.53 (5.21–21.36)** ^∗^
	Lightly active	1.42 (0.75–2.71)	**6.13 (1.042–16.00)** ^∗^
	Moderate and highly active	1	1

Menopause status	Premenopause	0.59 (0.34–1.02)	
	Postmenopause	1	

Chest radiation therapy	Yes	6.07 (2.81–13.08)^∗∗^	**6.43 (3.20–13.90)** ^∗^
	No	1	1

Age of menarche	≤15 years	3.68 (0.40–4.15)	
	>15 years	1	

Use modern of contraceptive	Yes	0.80 (0.45–1.42)	
	No	1	

Physical activity ∗ duration of breastfeeding	Sedentary ∗ duration of breastfeeding	0.79 (0.37−0.17)^∗∗^	0.13 (0.01–2.01)
	Lightly active ∗ duration of breastfeeding	0.05 (0.20−0.15)^∗∗^	0.14 (0.01–2.86)
	Moderate and highly active	1	

Chest radiation therapy ∗ duration of breastfeeding	Yes	0.09 (0.05–0.19)^∗∗^	0.72 (0.4–11.69)
	NO	1	

AIC			174.176

*Note.* ^∗∗^Denotes *P* < 0.05, ^∗^Denotes *P* < 0.25 for bivariate analysis. Boldface highlights results that are statistically significant at 0.05 (*P* < 0.05) in the multivariable analysis.

## Data Availability

The datasets analyzed during the current study can be made available from the corresponding author upon reasonable request. Researchers or individuals interested in accessing the data may reach out to the corresponding author for further information.

## Abbreviations

abrAOR: Adjusted odds ratio
BMI: Body mass index
CI: Confidence interval
COR: Crude odds ratio
FHCSH: Felege Hiwot Comprehensive Specialized Hospital.
